# Marine heatwave causes unprecedented regional mass bleaching of thermally resistant corals in northwestern Australia

**DOI:** 10.1038/s41598-017-14794-y

**Published:** 2017-11-03

**Authors:** Morane Le Nohaïc, Claire L. Ross, Christopher E. Cornwall, Steeve Comeau, Ryan Lowe, Malcolm T. McCulloch, Verena Schoepf

**Affiliations:** 1 ARC Centre of Excellence for Coral Reef Studies, UWA Oceans Institute and School of Earth Sciences, The University of Western Australia, Perth, WA Australia; 2 The Western Australian Marine Science Institution, Perth, WA Australia

## Abstract

In 2015/16, a marine heatwave associated with a record El Niño led to the third global mass bleaching event documented to date. This event impacted coral reefs around the world, including in Western Australia (WA), although WA reefs had largely escaped bleaching during previous strong El Niño years. Coral health surveys were conducted during the austral summer of 2016 in four bioregions along the WA coast (~17 degrees of latitude), ranging from tropical to temperate locations. Here we report the first El Niño-related regional-scale mass bleaching event in WA. The heatwave primarily affected the macrotidal Kimberley region in northwest WA (~16°S), where 4.5–9.3 degree heating weeks (DHW) resulted in 56.6–80.6% bleaching, demonstrating that even heat-tolerant corals from naturally extreme, thermally variable reef environments are threatened by heatwaves. Some heat stress (2.4 DHW) and bleaching (<30%) also occurred at Rottnest Island (32°01’S), whereas coral communities at Ningaloo Reef (23°9’S) and Bremer Bay (34°25’S) were not impacted. The only other major mass bleaching in WA occurred during a strong La Niña event in 2010/11 and primarily affected reefs along the central-to-southern coast. This suggests that WA reefs are now at risk of severe bleaching during both El Niño and La Niña years.

## Introduction

Coral reefs are in serious decline worldwide due to a combination of increasing local and global anthropogenic pressures^1,2^. Rising atmospheric CO_2_-concentrations are causing ocean warming, which leads to more intense and frequent mass coral bleaching events. To date, three global mass bleaching events (1998, 2010, and 2015/16) have been documented since the 1980s and were associated with El Niño-Southern Oscillation (ENSO) driven warming events^3–5^, highlighting the sensitivity of corals to climate-driven marine heatwaves^6^. Bleaching most commonly occurs during periods of thermal stress when corals lose their algal dinoflagellate symbionts (*Symbiodinium* spp.), resulting in a pale or white appearance of the coral colony^7–9^. Given that the majority of scleractinian corals meet most of their metabolic demand from carbon derived from symbiont photosynthesis^10^, bleaching results in severe resource limitation and thus significantly weakens them. While bleached corals can sometimes recover, the physiological damage caused during bleaching often results in extensive coral mortality^11–13^. Therefore, warming-related mass bleaching events are among the greatest threats to coral reefs today^5,14^. Since these events can lead to mass mortality from regional to global scales, they impact both the diversity and functioning of coral reef ecosystems^15^, and also threaten the socio-economic services on which millions of people worldwide depend^16^.

In 2015/16, unusually high ocean temperatures associated with one of the strongest El Niño events on record triggered an unprecedented global coral reef crisis, initiating what would become the third documented global mass bleaching event^17^. This event was estimated to have impacted 38% of the world’s coral reefs and became the longest and most severe mass bleaching event on record^5,17^. It has devastated coral reefs across all three major ocean basins^3,5,11,18–20^, and caused annually recurring mass bleaching in several locations for the first time^3,21^. Coral reefs in the South China Sea, for example, experienced unprecedented mass bleaching with 40% coral mortality in 2015^11^, while in the Maldives live coral cover declined by 75% due to severe bleaching in 2016^19^. Similarly, the Great Barrier Reef experienced the worst bleaching event in its history in 2016^5^, followed by another severe bleaching event just one year later^21^.

In late 2015, the U.S. National Oceanic and Atmospheric Administration (NOAA)’s Coral Reef Watch predicted significant coral bleaching and/or mortality (alert levels 1 and 2) for most coral reefs in Western Australia (WA) during the austral summer 2016^17^. NOAA’s bleaching forecasts showed that the greatest heat stress would occur along the northern WA coast, particularly in the remote Kimberley region. The macrotidal Kimberley region is one of the most extreme natural coral reef environments in the world, with tidal ranges up to 12 m, turbid waters associated with strong tidal currents and terrestrial sediment discharge, and sea surface temperatures (SST) exceeding 30 °C for five months per year^22–25^. Despite these extreme environmental conditions, highly diverse coral reefs exist throughout the Kimberley^25^. Interestingly, the highly fluctuating temperatures of intertidal reef habitats (up to 7 °C daily) have been shown to enhance coral thermal tolerance^26^, consistent with other work on thermally variable reef environments^27,28^. However, Kimberley corals were nevertheless not immune to severe heat stress simulated in a tank experiment^26^, raising the question how they would respond to periods of elevated temperatures associated with marine heat waves.

**Figure 1:**
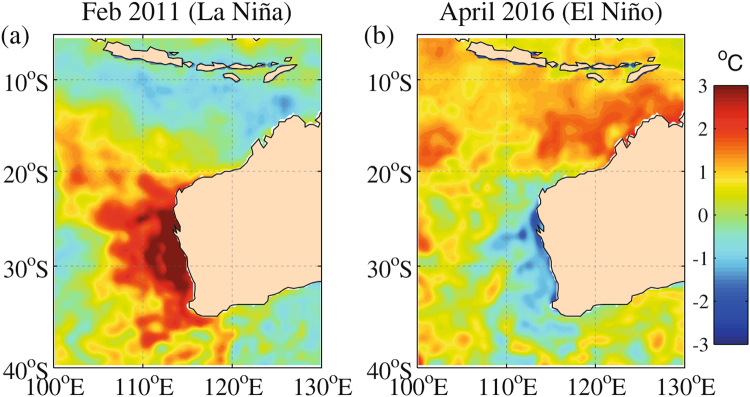
Large-scale patterns of sea surface temperature (SST) anomalies within the Eastern Indian Ocean along Western Australia, monthly-averaged for the periods when SST anomalies were near maximum: (**a**) Feb 2011 during the La Niña; and (**b**) Apr 2016 during the El Niño. SST anomalies are based on NOAA 1/4° daily optimally interpolated SST version 2 (OISST V2) relative a 1971–2000 climatological mean for the respective month using data available from https://www.ncdc.noaa.gov/oisst/data-access ^63^. Map figures are plotted using MATLAB R2015b (http://www.mathworks.com/).

**Figure 2:**
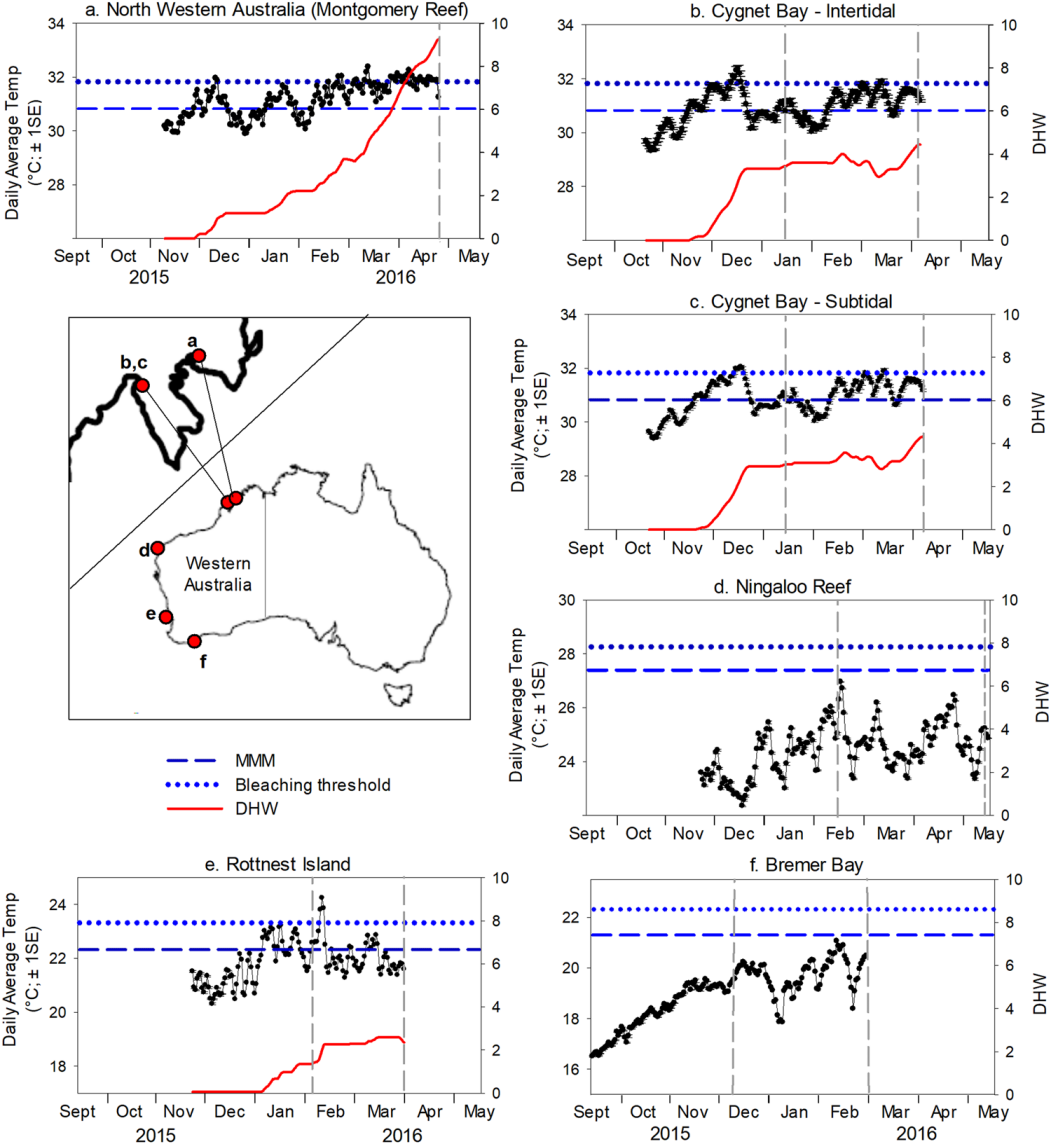
Map of Western Australia showing the locations of all survey sites and corresponding sea surface temperature (SST) plots for (**a**) Montgomery Reef, (**b**) Cygnet Bay Intertidal, (**c**) Cygnet Bay Subtidal, (**d**) Ningaloo Reef, (**e**) Rottnest Island, and (**f**) Bremer Bay. SSTs are in situ temperature data for all sites except Montgomery Reef, for which satellite-derived data were used (NOAA Coral Reef Watch 5-km virtual station North Western Australia). SSTs are shown for the 12 weeks prior to the first survey time point through to the second survey time point at each site. Dashed vertical lines indicate survey time points. MMM = maximum monthly mean, bleaching threshold = local MMM + 1 °C, DHW = Degree Heating Weeks (see Methods for more details). Line art imagery created in Adobe Illustrator (version CS6) based on a map image created in R software (version 3.4.0, R Core Team, 2017, https://www.R-project.org) by S. Comeau.

To date, regional-scale mass bleaching (i.e. extending 100 s of kilometres) has been documented in WA only once during a strong La Niña event in 2010/11^29–33^, but not during strong El Niño years (e.g. 1997/98, 2010) that caused mass bleaching in many other locations around the world. Although some offshore oceanic coral atolls in northern WA (e.g. Scott Reef) did bleach severely in 1998^34^, the coastal WA region has largely been considered to be at low risk from bleaching during strong El Niño events. During the La Niña-driven heatwave in 2010/11, sea surface temperatures exceeded normal summer temperatures by an average of 3 °C along the WA coast between 22°S (Ningaloo Reef) and 34°S (Cape Leeuwin)^33^ (Fig. 1a). This resulted, for example, in 12–100% bleaching at the Houtman Abrolhos Islands^29,35^ and 79–92% at Ningaloo Reef^12^. However, coral reefs in northern WA, including the Kimberley, escaped this marine heatwave and associated bleaching. Early forecasts in late-2015 by NOAA’s Coral Reef Watch of an impending heatwave during the austral summer 2016 raised significant concern for northern WA, given that regional-scale mass bleaching has never occurred in WA during a strong El Niño. As a consequence we conducted extensive coral health surveys at five sites in four different bioregions along the WA coast between December 2015 and May 2016 (Fig. 2), both prior to and following the peak warming. Since El Niño events will likely increase in frequency and intensity due to climate change^36^, understanding how these extreme climatic events impacts coral reefs in WA is critical to predict their persistence under continued ocean warming and climate change.

## Methods

Underwater coral health surveys were conducted at five sites in four different bioregions along the WA coast, spanning ~2900 km and ~17 degrees of latitude, and ranging from a tropical climate in the north to a temperate climate in the south. From north to south, these sites/bioregions were Montgomery Reef and Cygnet Bay (Kimberley region), Coral Bay (Ningaloo Reef), Rottnest Island (subtropical Southwest), and Bremer Bay (temperate Southwest) (Fig. 2). Coral health was assessed at all sites except Montgomery Reef prior to and during the predicted heat stress period between December 2015 and May 2016 (Table 1).

**Table 1:** Coral health survey methodology and survey dates for each site. N/A = not applicable.

Region	Kimberley Region			Ningaloo Reef	Subtropical Southwest	Temperate Southwest
Site	Montgomery Reef	Cygnet Bay Subtidal	Cygnet Bay Intertidal	Coral Bay	Rottnest Island	Bremer Bay
Type of survey	15 m long transects; photoquadrat 50 × 50 cm every 0.5 m	15 m long transects; photoquadrat 50 × 50 cm every 0.5 m	15 m long transects; photoquadrat 50 × 50 cm every 0.5 m	15 m long transects; photoquadrat 50 × 50 cm every 1 m	15 m long transects; photoquadrat 50 × 50 cm every 0.5 m	30 min roving diver surveys; colony counts/ photos of *Turbinaria*
First survey time point	N/A	13–17 January 2016	13–17 January 2016	15 February 2016	5 February 2016	8–9 December 2015
Number of transects/measured colonies	N/A	6 transects	7 transects	6 transects	7 transects	45 colonies
Second survey time point	23 April 2016	6–9 April 2016	6–9 April 2016	17 May 2016	1 April 2016	1–3 March 2016
Number of transects/measured colonies	4 transects	6 transects	6 transects	6 transects	6 transects	78 colonies

### Survey sites

#### Kimberley region (Montgomery Reef, Cygnet Bay)

Surveys in the macrotidal Kimberley region were conducted at remote Montgomery Reef (15°59′33.29″S, 124°4′9.34″E; Fig. 2), which is Australia’s largest inshore reef (380 km^2^). The tidal range in this area is up to 12 m during spring tide, resulting in vast areas of the reef being regularly exposed to air and forming a large plateau at low tide. While the plateau itself is dominated by coralline algae, seaweed and solitary corals, diverse hard coral communities are found along the slopes of the plateau, consisting mostly of sub-massive corals^37^. Due to the remote location of Montgomery Reef, surveys in the shallow subtidal were conducted only in April 2016 when severe bleaching was detected at Cygnet Bay ~120 km to the southwest (Fig. 2). These surveys were conducted concurrently with extensive aerial surveys of the region^5^. *In situ* temperature data are not available for Montgomery Reef, but seasonal monthly temperatures generally range from ~25.5 °C to ~31 °C in the region, with a long-term maximum monthly mean (MMM) sea surface temperature (SST) of 30.827 °C^38^.

Surveys were also conducted in the subtidal and intertidal environments at Shell Island, Cygnet Bay (16°28′46.8″S, 123°2′36.6″E; Fig. 2) in January and April 2016. These environments are described in detail elsewhere^24,26^. The spring tidal range in Cygnet Bay is ~8 m. At low tide, the intertidal represents a shallow tide pool with a slack water period of several hours, resulting in extreme temperature fluctuations (up to 7 °C daily, maxima of up to 37 °C) and regular aerial exposure of shallow corals. In contrast, the subtidal zone experiences more moderate temperatures where aerial exposure of coral occurs only a few days per year during extreme low tides^24^. Branching *Acropora* colonies dominate coral cover in both environments. *In situ* seasonal temperatures in both environments are similar and range from ~22.0 °C to 31.5 °C (daily averages)^24^. Bleaching thresholds for both intertidal and subtidal corals were previously experimentally established to be ~32 °C, ~1 °C above the local MMM^26^. We therefore used the long-term MMM of 30.827 °C from NOAA’s 5-km virtual station North Western Australia^38^ for our analyses.

#### Ningaloo Reef (Coral Bay)

Surveys were conducted at Coral Bay (23°9′21.47″S, 113°45′7.79″E; Fig. 2) located in the southern section of Ningaloo Reef, which is Australia’s largest fringing reef encompassing over 5000 square kilometers of ocean. Surveys were conducted on the reef flat during February and May 2016, at a depth of ~2 m. Coral cover at this site primarily consists of branching *Acropora* corals^12^. Seasonal monthly temperatures range from ~22–23 °C to ~27–28 °C^39^. We used the NOAA long-term MMM of 27.399 °C for our analyses^40^.

#### Subtropical Southwest (Rottnest Island)

Rottnest Island is located ~20 km off the coast of Perth in southwestern Australia (32°01′00.4″S, 115°31′06.0″E; Fig. 2), and is characterized by a combination of subtropical and temperate marine habitats due to the influence of the Leeuwin Current, which transports warm oligotrophic water poleward along the Western Australian coastline. These waters support over 25 species of scleractinian corals at this high-latitude location^41^. Surveys were conducted in February and April 2016 at a depth of ~1–2 m in Salmon Bay where coral communities are dominated by *Acropora* and *Pocillopora* ^42^. This is the southernmost documented occurrence of the genus *Acropora* in WA. *In situ* seasonal temperatures in 2013–2014 ranged from ~18 °C to 23 °C^42^. We used the NOAA long-term MMM of 22.323 °C for our analyses^43^.

#### Temperate Southwest (Bremer Bay)

Bremer Bay is located on the southern coast of WA, ~400 km southeast of Perth (Fig. 2), and features extensive high-latitude coral communities dominated by *Turbinaria reniformis* colonies growing at the geographical limits of their distribution in WA. Surveys were conducted in December 2015 and March 2016 at a depth of ~8 m at both Back Beach (34°25′16.57″S, 119°23′35.43″E) and Little Boat Harbour (34°28′8.50′S, 119°21′34.09″E). *In situ* seasonal temperatures range from ~16 °C to ~21 °C, with a MMM of 21.3 °C (historical data records obtained from the Bremer Bay “888 Abalone” farm located ~20 m from Back Beach for August 2009 to December 2014 were combined with *in situ* temperature data from December 2014 onwards).

### Coral health surveys and sea surface temperature monitoring

The following methodology was used for surveys at all sites except Bremer Bay (see Table 1 for details). Four to six 15 m transects were conducted at randomized locations via intertidal walking or snorkeling. Care was taken to ensure that all transects were conducted at a similar depth. High-resolution photos of a 50×50 cm quadrat were taken every 0.5–1 m along the transect line. At Bremer Bay, a different methodology was used due to the sporadic coral cover at this high-latitude site. Divers conducted roving diver surveys^44,45^, photographing and scoring any coral colonies that they encountered within a 30 min time period.

HOBO U22 v2 temperature loggers (±0.2 °C; Onset Computer Corp) were deployed at each site except the remote Montgomery Reef, and continuously recorded *in situ* water temperature every 15 minutes during the study period (i.e. from 22 October 2015 to 06 April 2016 at Cygnet Bay, from 24 November 2015 to 17 May 2016 at Coral Bay, from 14 November 2015 to 01 April 2016 at Rottnest Island, and 16 September 2015 to 01 March 2016 at Bremer Bay). These time periods were chosen to assess heat stress during the 12 weeks prior to the first survey time point and between the first and second survey time point. For Montgomery Reef, satellite-derived SSTs from NOAA’s 5-km virtual station North Western Australia were used for a similar time period as at nearby Cygnet Bay (9 November 2015 to 23 April 2016). To assess cumulative heat stress, degree heating days (DHD)^46^ were calculated as the sum of all positive temperature anomalies (i.e. daily average SST exceeding the local MMM) over the previous 12 weeks; this was found to provide more realistic estimates of heat stress than NOAA’s methodology of accumulating for only positive temperature anomalies ≥1 °C (see Discussion). Large DHD values were converted to Degree Heating Weeks (DHW) by dividing by 7. The bleaching threshold was set at 1 °C above the local MMM, which is generally thought to be the threshold for bleaching in most coral species^47,48^.

### Photoquadrat analysis

Photoquadrats were analyzed using the software photoQuad^49^ by one person to keep observer bias equal across all photos. The outline of the quadrat within the photo was manually defined. Substrate type was defined using stratified random point counts (100 points), such that the spawn canvas was divided into sub-cells and points spawned within each cell in a random manner. This method ensures that at least one point is present in each sub-cell. The following types of substrate were distinguished: hard coral, soft coral, seaweed/seagrass/encrusting coralline algae/turf, sand/rubble, rock, and unknown. The ‘unknown’ category was applied to quadrat areas that could not be unequivocally assigned to a substrate category; this was primarily the case for Kimberley surveys where high water turbidity and rapidly changing water levels created challenging optical conditions. Hard corals were identified to genus level when possible and further assigned a morphology (i.e. branching, plate-like/plating, encrusting and mounding/sub-massive/massive). Each coral colony was scored using the following four health categories as a categorical bleaching score^13^: unbleached (UB), moderately bleached (M: <50% of the colony bleached or colony pale), severely bleached (S: >50% bleached), and dead (D) (see Supplementary Figure S1).

### Statistical analysis

Since survey methodology differed between Bremer Bay and the other sites, different statistical tests were used to analyse the data. For all sites except Bremer Bay, count data from the photoquadrat analyses were converted to percent abundance data and square root transformed prior to multivariate statistical analysis. The variability of the four health categories (UB, M, S and D) for each coral genus was statistically tested for differences between sites and time periods using Permutational Multivariate Analyses of Variance (PERMANOVAs), with transects as replicates, the Bray-Curtis similarity index and 9999 iterations. Several PERMANOVAs were conducted to accommodate the fact that not all sites were surveyed at all time points (i.e. Montgomery Reef), and that one site (Cygnet Bay) had two different environments (intertidal and subtidal) where coral communities are known to differ in their heat tolerance^26^:

1. For sites that were surveyed before and during the peak heat stress, a two-way PERMANOVA was conducted to test the effects of time (2 levels) and site (4 levels: Cygnet Bay intertidal, Cygnet Bay subtidal, Ningaloo Reef, Rottnest Island) on coral health across all genera.
2. To assess whether there were any differences between sites during the peak heat stress, a one-way PERMANOVA was conducted to test the effect of site (5 levels: Montgomery Reef, Cygnet Bay intertidal, Cygnet Bay subtidal, Ningaloo Reef, Rottnest Island) on coral health across all genera at the second time point only.
3. To assess whether intertidal and subtidal environments at Cygnet Bay differed in their response to heat stress, two-way PERMANOVAs were conducted to test the effects of time (2 levels) and environment (2 levels: intertidal and subtidal) on both coral health across all genera and the four health categories summarized for all hard corals.

Post-hoc pairwise comparisons were calculated using a sequential Bonferroni correction to adjust *p*-values. Principal Component Analyses (PCAs) were used to visualize these data.

For Bremer Bay, data from the roving diver surveys at Back Beach and Little Boat Harbour were pooled after a χ² test showed no statistically significant difference in coral health between the two locations for both December 2016 and March 2016 (Table 2). A second χ² test was then used to test whether coral health differed between these two time points. All analyses were conducted using the software PAST version 3.12^50^. *P*-values ≤ 0.05 were considered significant. The raw data for this manuscript will be submitted to the Pangaea Database after publication.

**Table 2:** Results of statistical analyses to compare coral health between sites and time points.P-values** ≤ **0.05 are highlighted in bold. Df = degrees of freedom; N/A = not applicable.

Multivariate analyses	Factor	*df*	*F*-value	*p*-value	Post-hoc pairwise comparisons (*p*-values)
Two-way PERMANOVA across all coral genera for all sites except Montgomery and Bremer Bay at two time points	Time	1	10.9	**<0.001**	N/A*
	Site	3	10.0	**<0.001**	
	Interaction	3	4.6	**<0.001**	
One-way PERMANOVA across all coral genera for all sites except Bremer Bay only at the second time point	Site	4	11.6	**<0.001**	**<0.05** for all sites
Two-way PERMANOVA across all coral genera for Cygnet Bay only, subtidal vs. intertidal, at two time points	Time	1	24.7	**<0.001**	N/A*
	Site	1	2.8	0.09	
	Interaction	1	1.2	0.08	
Two-way PERMANOVA as above but for the health categories summarized for all coral genera	Time	1	1.7	0.20	N/A*
	Site	1	194.6	**<0.001**	
	Interaction	1	6.3	**0.01**	
**Bremer Bay statistical analyses**		**Back Beach**		**Little Boat Harbour**	
		**December 2015**	**March 2016**	**December 2015**	**March 2016**
Number of sampled colonies		20	55	25	23
		**χ² value**		***p*** **-value**	
	***df***	**December 2015**	**March 2016**	**December 2015**	**March 2016**
χ² test: difference between Back Beach and Little Boat Harbour	1	1.6	0.5	0.2	0.5
χ² test: difference between December 2015 and March 2016 with pooled data	1	0.05		0.8	

^*^Note that post-hoc pairwise comparisons are not available for two-way PERMANOVAs in PAST.

## Results

### Sea surface temperatures and heat stress

Substantial heat stress occurred in the Kimberley region (i.e. Montgomery Reef, Cygnet Bay) and, to a lesser degree, in the subtropical Southwest (i.e. Rottnest Island) during the study period (Figs 1 and 2). Montgomery Reef was at highest risk of bleaching, with daily-averaged SSTs exceeding the local MMM for 113 days from 9 November 2015 to 23 April 2016, resulting in 64.9 DHD (9.3 DHW) by late April 2016 when surveys were conducted (Fig. 2a). Similarly, daily-averaged SSTs in the intertidal and subtidal environments at nearby Cygnet Bay exceeded the local MMM for 99 and 95 days, respectively, between 22 October 2015 and 6 April 2016, and by early April 2016 accumulated to 4.5 and 4.3 DHW, respectively (Fig. 2b,c). However, heat stress from mid-November 2015 onward had already reached ~3 DHW by the time the surveys were conducted in mid-January 2016. At Rottnest Island, SSTs exceeded the local MMM for 39 days between 14 November 2015 and 1 April 2016, resulting in 16.4 DHD (2.4 DHW) by the second survey (Fig. 2e). However, some heat stress had already occurred throughout January 2016, resulting in 1.4 DHW at the first survey. In contrast, SSTs at Ningaloo Reef and Bremer Bay did not exceed the local MMM on any day during the respective study periods (Fig. 2d,f).

### 2015/16 coral bleaching along the WA coastline

**Figure 3:**
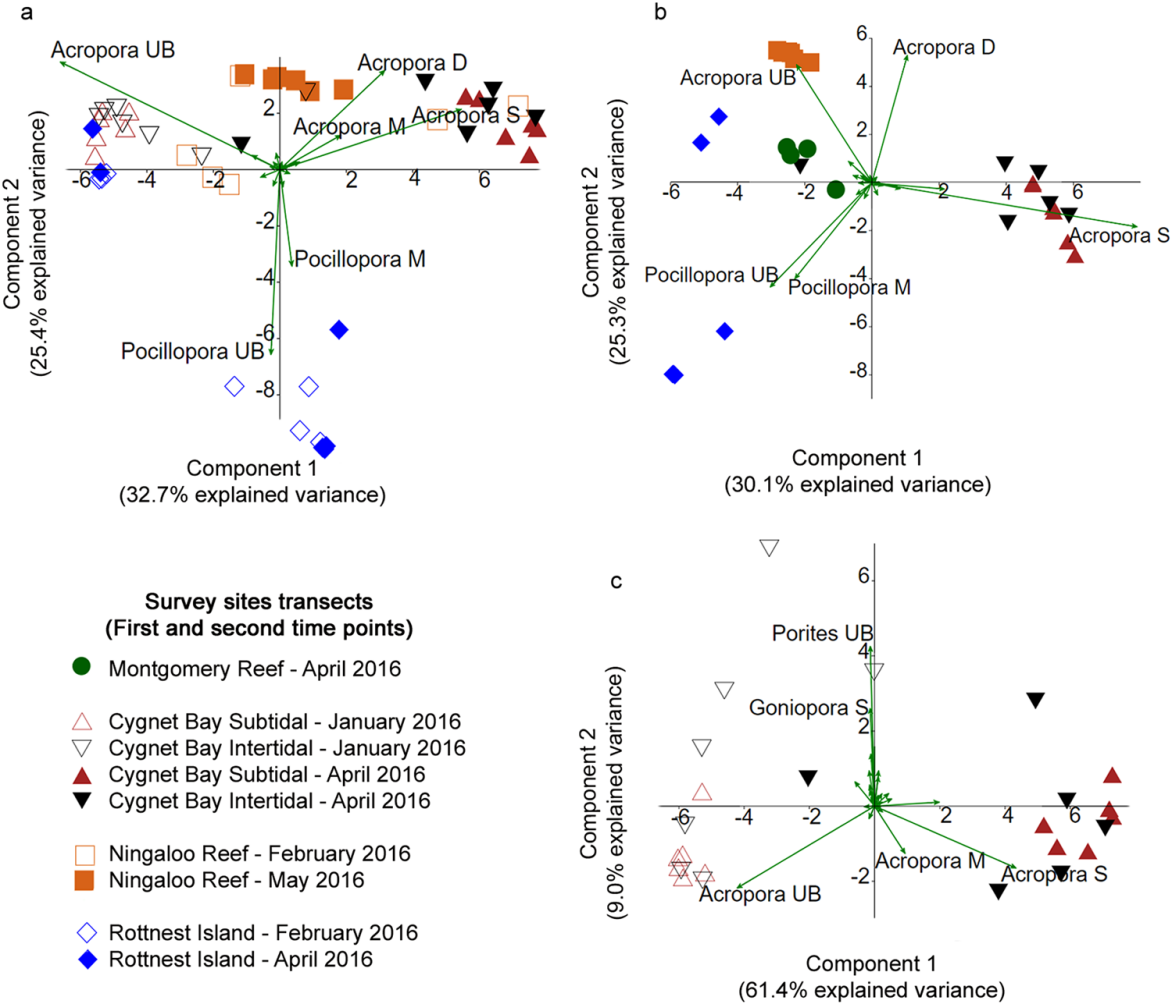
Principal components analysis (PCA) of coral health across all hard coral genera for (**a**) all sites that were surveyed at the two time points, (**b**) all sites (except Bremer Bay) at the second time point only, and (**c**) Cygnet Bay only at the two time points to compare coral health in the intertidal and subtidal environments. Bremer Bay was not included in these analyses due to the different survey methodology (see Methods). Vectors represent specific coral genera and their associated health status that had the greatest influence on overall coral health. Symbols represent individual transects. UB = unbleached, M = moderately bleached, S = severely bleached, D = dead.

**Figure 4:**
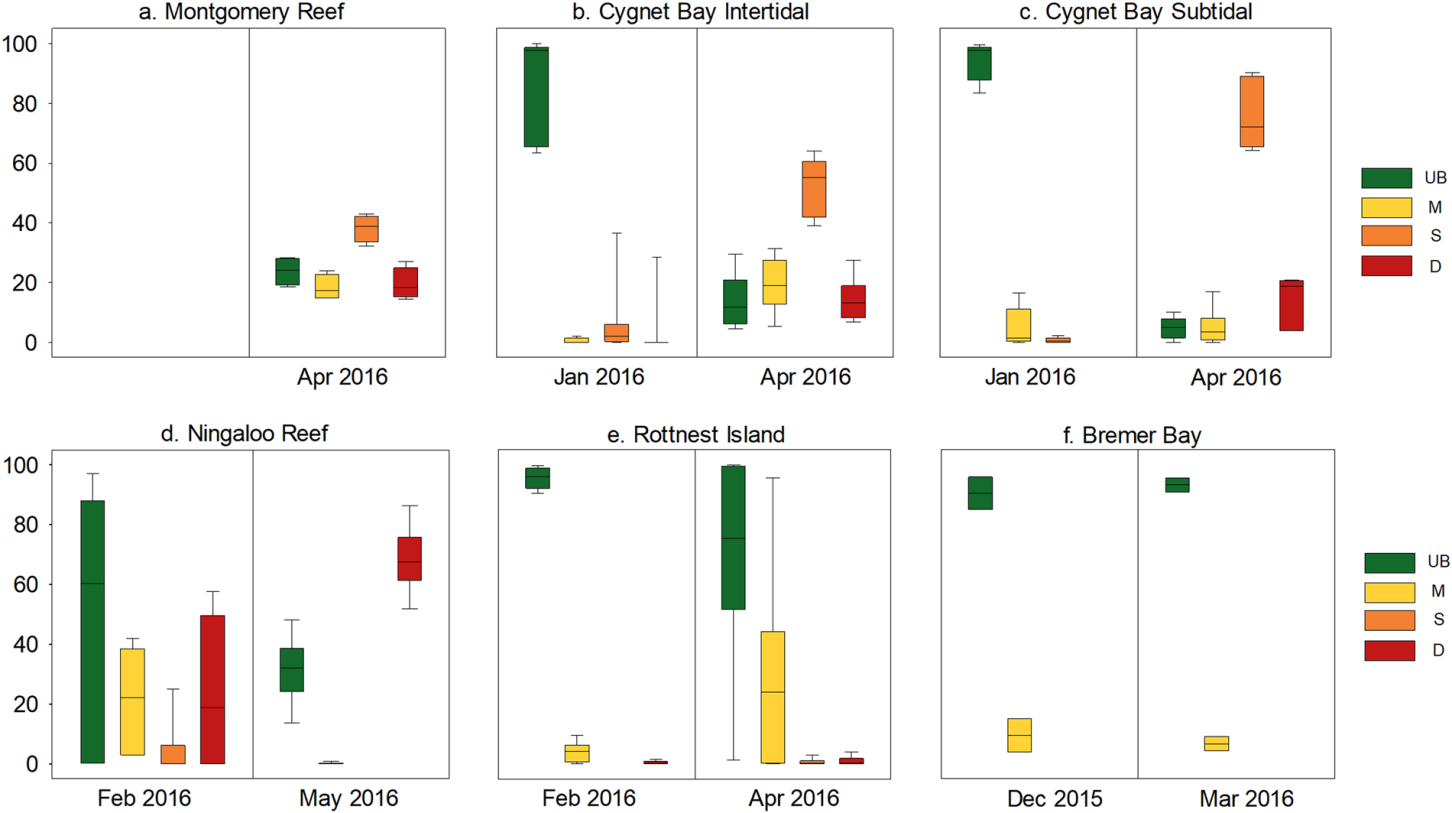
Box plots showing percent cover of the 4 health categories summarized for all hard coral genera at (**a**) Montgomery Reef, (**b**) Cygnet Bay Intertidal, (**c**) Cygnet Bay Subtidal, (**d**) Ningaloo Reef, (**e**) Rottnest Island, and (**f**) Bremer Bay at the first and the second survey time point. The four health categories are unbleached (UB) in green, moderately bleached (M) in yellow, severely bleached (S) in orange, and dead (D) in red. The first survey time points are in December (Dec) 2015, January (Jan) or February (Feb) 2016 and the second survey time points are in March (Mar), April (Apr) or May 2016. Box plots show median and 25^th^ and 75^th^ percentiles, with whiskers (error bars) indicating the 10^th^ and 90^th^ percentile.

Initially in January 2016, no major bleaching was observed at any of the sites in the Kimberley; however, severe bleaching was observed at all Kimberley sites in April 2016. Some bleaching was observed at Rottnest Island in May 2016, whereas Ningaloo Reef and Bremer Bay did not experience major bleaching at either time point (Figs 3, 4). These results are consistent with patterns of heat stress across WA (Fig. 2). However, the site at Ningaloo Reef experienced high levels of coral mortality throughout the study period, despite no evidence of heat stress (Figs 1, 2d).

#### Sites that were surveyed before and during the predicted peak heat stress

At Cygnet Bay, Ningaloo Reef and Rottnest Island, a significant interactive effect of site and time was observed for coral health across all coral genera (Table 2). This was consistent with the PCA plot (Fig. 3a), where transects conducted prior to and during the predicted peak heat stress were clearly separated along principal component 2, driven by differences in the abundance of unbleached versus bleached *Acropora* corals. Transects conducted at Ningaloo Reef at the second survey time clustered along the vector for dead *Acropora*. Rottnest Island was clearly distinct from the other sites, particularly prior to the peak heat stress, due to unbleached *Pocillopora* contributing substantially to live coral cover.

These statistical findings were confirmed by trends in coral health categories summarized for all hard coral genera (Fig. 4, Table 3). Prior to the predicted peak heat stress, most corals were unbleached in the intertidal (88.6 ± 4.1% (mean ± standard error)) and subtidal (94.3 ± 2.6%) at Cygnet Bay, although some bleached and dead corals were observed (Fig. 4b,c). Similarly, most corals were unbleached at Rottnest Island (95.8 ± 1.3%, Fig. 4e). Coral cover at Ningaloo Reef was also dominated by unbleached corals (50.6 ± 17.1%, Fig. 4d), but with a high percentage of moderately bleached and dead corals compared to the other sites (21.6 ± 6.9 and 23.7 ± 10.9%, respectively). Three months later during the peak of heat stress, severely bleached corals dominated coral cover at Cygnet Bay, although there were more severely bleached corals in the subtidal (75.6 ± 4.8%) than intertidal (52.6 ± 4.0%). In contrast, the majority of corals at Rottnest Island were still unbleached (70.0 ± 14.8%) but moderately bleached corals increased from 3.7 ± 1.3% to 28.5 ± 14.3%. At Ningaloo Reef, unbleached corals decreased in abundance (31.5 ± 4.7%) and dead corals now dominated coral cover (68.3 ± 4.6%).

**Table 3:** Abundance of the most common hard coral genera and associated health categories at all study sites during the first and second survey time point.

Site		Most common genera	Most abundant health category	
			Dec. 2015–Feb. 2016	April/May 2016
**Montgomery Reef**		*1*. *Montipora* (>29%)	N/A	10.8 ± 2.5% severely bleached *Montipora*
		2. *Acropora*, *Galaxea*, *Platygyra*, and *Favites*		
**Cygnet Bay**	Intertidal	*1*. *Acropora* (>71%)	65.6 ± 12.1% unbleached *Acropora*	41.3 ± 9.3% severely bleached *Acropora*
		*2*. *Porites*, *Goniopora*, *Lobophyllia*, and *Galaxea*		
	Subtidal	*1*. *Acropora* (>87%)	87.2 ± 5.2% unbleached *Acropora*	69.7 ± 5.6% severely bleached *Acropora*
		*2*. *Porites*, *Turbinaria*, *Favites*, and *Seriatopora*		
**Ningaloo Reef**		*1*. *Acropora* (>61%)	23.7 ± 10.9% dead *Acropora*	66.6 ± 4.9% dead *Acropora*
		*2*. *Favites*, *Platygyra*, *Porites*, and *Dipsastraea*		
**Rottnest Island**		*1*. *Pocillopora* (>51%)	46.7 ± 15.8 unbleached *Pocillopora*	37.4 ± 15.6% unbleached *Pocillopora*
		*2*. *Acropora*, *Platygyra*, and *Favites*		
**Bremer Bay**		*Turbinaria reniformis*	41 unbleached colonies	72 unbleached colonies

Data represent the mean percentage ( ± SE) of total live coral cover. N/A = not applicable.

#### Comparing all sites (except Bremer Bay) during predicted peak heat stress

During the predicted peak heat stress in April 2016, all sites (excluding Bremer Bay) differed significantly from one another (Table 2). This pattern was also evident in the PCA plot (Fig. 3b), with Cygnet Bay transects having a large cover of severely bleached *Acropora*, particularly in the subtidal. Montgomery Reef represented a separate cluster due to coral cover generally being dominated by *Montipora* rather than *Acropora* and *Pocillopora* (Table 3). A large percentage (38.2 ± 2.5%) of the total coral cover at Montgomery Reef was severely bleached in April 2016 (Fig. 4a). In contrast, the typical coral cover at sites with no major bleaching was driven by unbleached corals (Fig. 3b and Fig. 4d,e). At Rottnest Island, unbleached *Acropora* and *Pocillopora* dominated coral cover, whereas unbleached *Acropora* dominated cover at Ningaloo Reef, despite there also being a significant percentage of dead corals (Fig. 4d,e).

#### Comparing intertidal and subtidal environments at Cygnet Bay

Coral health across all genera differed significantly between January and April 2016 at Cygnet Bay, but not between intertidal and subtidal environments (Table 2, Fig. 3c). However, when the same analysis was conducted with coral health pooled for all coral genera, a significant interactive effect of time and site was observed (Table 2). This is consistent with the substantially greater percentage of severely bleached corals in the subtidal (75.6 ± 4.8%) than intertidal (52.6 ± 4.0%) in April 2016 (Fig. 4b,c).

#### Changes in coral health at Bremer Bay

At the temperate site in Bremer Bay, a total of 123 *T*. *reniformis* colonies ranging from 2–150 cm in diameter were surveyed in December 2015 (*n* = 45) and March 2016 (*n* = 78). At both survey times, more than 90% of the surveyed colonies were unbleached (Table 3), with the remaining corals being moderately bleached (*n* = 4 in December, *n* = 6 in March; Fig. 4). Therefore, coral health was not significantly different between the two time points (Table 2).

Further details about the health status of each coral genus at the different survey time points and sites are provided in Supplementary Table S1 online.

## Discussion

We show here that marine heatwaves associated with extreme climatic events such as the record-strength 2015/16 El Niño have the potential to cause unprecedented regional-scale mass bleaching, even in coral reef regions that harbour naturally heat-resistant corals that have escaped mass bleaching in previous El Niño years. This occurred in the macrotidal Kimberley region in northwestern Australia during the austral summer of 2016, where highly diverse and naturally stress-resistant coral reef communities have been observed to thrive under conditions that corals from more typical reef environments would usually not survive (e.g. long aerial exposure, daily temperature fluctuations of up to 7 °C and temperature maxima of up to 38 °C during low tide^24,26,51^). A recent study showed that these highly fluctuating temperatures enhance the thermal tolerance of Kimberley corals^26^. Nevertheless, Kimberley coral reefs experienced unprecedented mass bleaching in April 2016 in response to severe heat stress (~4–9 DHW, Fig. 2), with more than 56% and 71–80% of live coral cover being bleached at our two Kimberley study sites (Montgomery Reef and Cygnet Bay, respectively) (Fig. 4). In addition, broader-scale aerial surveys of 25 reefs in the southern Kimberley, conducted over the same time period, further confirmed the regional scale of this mass bleaching event and showed that most reefs in this region had 30–60% bleaching^5^. This demonstrates that even naturally heat-resistant corals from extreme temperature environments such as the Kimberley region are not immune to marine heatwaves and extreme climatic events. These findings are consistent with experimental work on Kimberley corals showing that heat stress equivalent to ~3 DHW resulted in severe bleaching and mortality, although heat stress in that study was applied over a much shorter time period (<2 weeks)^26^. Coral communities in other naturally extreme temperature environments, such as the Persian/Arabian Gulf where corals have the world’s highest known bleaching thresholds (~35–36 °C), are also not immune to severe heat stress and have suffered from multiple episodes of bleaching associated with significant mortality over the last three decades^52^. This suggests that even naturally heat-resistant corals are significantly threatened by periods of sustained ocean warming, as it is currently unclear whether they can increase their heat tolerance over the time scales required to cope with future climate change.

The marine heatwave causing the 2016 mass bleaching in the Kimberley was characterized by long-lasting exposure to small positive temperature anomalies that rarely exceeded the local MMM by more than 1 °C (Fig. 2a–c). SSTs already rose above the local MMM in November 2015, resulting in increasing heat stress and DHW from that point onwards. As a consequence, some coral genera (i.e. *Seriatopora* and *Stylophora*) were already severely bleached in January 2016 but did not substantially influence overall coral community health due to their overall low abundance. Thus, Kimberley reefs experienced cumulative heat stress for ~5 consecutive months, demonstrating that even small positive temperature anomalies can cause severe bleaching and mortality when persisting over a long duration. The severity of the bleaching event was further confirmed by the significant bleaching of massive corals, which are typically more resistant to heat stress^53^.

Surprisingly, Montgomery Reef appeared to experience greater heat stress, yet a lower percentage of bleaching was observed compared to Cygnet Bay (Fig. 4a–c). This could be due to several factors, such as satellite-derived SSTs overestimating *in situ* heat stress, and/or coral cover being dominated by plate-like *Montipora* rather than *Acropora* corals (Table 3). Importantly, the choice of the MMM value can have a substantial influence on calculated heat stress and DHW values even when *in situ* temperature data are available. NOAA’s MMM of 30.827 °C (version 2) for the virtual station North Western Australia shows close agreement with experimentally established bleaching thresholds (MMM + 1 °C) of ~32 °C for Kimberley corals^26^, verifying the applicability of this MMM value for determining the bleaching threshold. However, NOAA’s climatology was recently updated (i.e. in 2017), emphasizing the challenge of accurately defining baseline temperatures that corals are adapted to. The latest version (i.e. version 3) now lists a much lower MMM (29.903 °C) for this station than version 2. This lower MMM value is most likely far too conservative because Kimberley corals do not show any signs of visible bleaching or declines in their photochemical efficiency (Fv/Fm) when exposed to daily average temperatures of ~31 °C for almost two weeks^26^. While we cannot exclude the possibility that Kimberley corals may have acclimatized to rising SSTs over the last few decades, these discrepancies highlight the importance of *in situ* temperature records and physiological data, especially in complex macrotidal reef environments that create particular challenges for satellite-derived SST monitoring.

The large spatial scale of the 2016 mass bleaching event in northern WA was, to the best of our knowledge, unprecedented. Kimberley offshore oceanic atolls (e.g. Scott Reef) have bleached previously^54,55^, and bleached again in 2016^5^; however, the vast inshore Kimberley region has escaped any bleaching prior to 2016, although it is possible that such events may have gone unnoticed or undocumented due to the remoteness of this region. The 2015/16 El Niño coincided with an extremely unusual and dry wet season in the Kimberley^56^, and also with the most extreme tides of the year. This likely resulted in increased temperature, light and UV stress as well as longer aerial exposure of shallow corals. Furthermore, the absence of major storms and cyclones would have prevented mitigation of both heat and light stress^57^. The combination of these factors most likely contributed or exacerbated heat stress^58^, thus resulting in unprecedented bleaching.

On finer spatial scales, the bleaching susceptibility of Kimberley coral communities differed significantly depending on small-scale differences in their thermal environment. During peak heat stress, coral health of subtidal coral communities at Cygnet Bay had declined significantly more than in intertidal communities, as indicated by a much higher percentage of severely bleached corals (Table 2, Fig. 4). This was the case despite similar exposure to heat stress (4.3 and 4.5 DHW in the subtidal and intertidal, respectively) and community composition (dominated by *Acropora* corals). These observations confirm experimental work showing that subtidal *Acropora* and *Dipsastraea* corals at Cygnet Bay have a lower thermal tolerance than their intertidal counterparts^26^. Although both intertidal and subtidal environments have similar average temperatures, they differ substantially with regard to daily temperature fluctuations and the frequency of aerial exposure during low tide^26^. Given that symbiont types did not differ between intertidal and subtidal corals^26^, our findings provide further evidence that extreme temperature fluctuations (up to 7 °C daily in the intertidal) represent a mechanism that enhances coral thermal tolerance^26–28^.

The 2016 mass bleaching event in the Kimberley region is the first such event to occur in WA during a strong El Niño year^32^ as regional-scale mass bleaching in WA extending over 100 s of kilometres has to date only occurred once during a strong La Niña year in 2010/11^29–33^. The 2010/11 heatwave primarily affected the central-to-southern WA coastal region (Fig. 1a), devastating coral communities from Ningaloo Reef to Rottnest Island. In the Ningaloo region, for example, the Exmouth Gulf had the highest amount of bleaching (~95%), whereas Coral Bay suffered ~25% bleaching^12^. In the Perth region, ~17% bleaching was observed^12^, which also extended to deeper reef communities at Rottnest Island (24–28 m)^59^. During the heatwave in 2016, satellite SST anomalies were negative (cooler) along the central-to-southern WA, which would suggest that no heat stress should occur (Fig. 1b). However, *in situ* temperature records showed that Rottnest Island experienced substantial heat stress (2.4 DHW, Fig. 2e). These reef-scale temperature anomalies are likely due to the positive local anomalies in air-sea heat fluxes that occurred over large scales across Western Australia at the time, which would have caused coastal warming in shallow reef waters off the coast of Perth^60^. This may explain why we observed 29.1% bleaching in Rottnest Island, although most corals were only moderately bleached (i.e. <50% bleached or colony pale) (Fig. 4). In contrast, no heat stress occurred in Coral Bay as the *in* situ SSTs stayed well below the local MMM for most of the summer period (Fig. 2d). We therefore consider it unlikely that the large proportion of dead corals observed at Coral Bay (Fig. 4), particularly in May 2016, was in some way related to unusual temperatures. Rather, we suspect that coral spawn slicks may have caused significant coral mortality at Coral Bay between our survey time points. Unusual weather conditions can trap coral spawn slicks in shallow bays, which then severely deplete oxygen concentrations in the water, resulting in mass mortality of coral and other reef organisms^61,62^. Such events have been documented at Coral Bay and other reefs in WA previously^61,62^. The high-latitude site Bremer Bay also did not experience any heat stress (Fig. 2f) and the small proportion of moderately bleached corals (Fig. 4f), thus, likely represents naturally lighter pigmentation due to seasonally higher light levels. Furthermore, it is unlikely that any heat stress occurred after early March given that SSTs generally peak in February at this site and remained below the MMM of 21.3 °C during all of March and April (C. Ross, unpublished data). To our knowledge, this site has not been surveyed during previous El Niño or La Niña years and it is therefore unclear whether it was affected by the marine heatwave in 2010/11.

This study shows that the geographic footprint of the 2010/11 and 2016 mass bleaching events in WA differed substantially (Fig. 1). While the 2010/11 event affected a larger geographic area, impacting coral reefs across 12° of latitude along the WA coast^30^, the 2016 event was regionally restricted to the northwestern parts of the state (although it should be noted that heat stress and bleaching occurred throughout northern Australia, including the Northern Territory and the Great Barrier Reef^30,32^). The 2010/11 event also coincided with a very active cyclone season^30^, which may have mitigated bleaching impacts to some extent^57^, whereas the opposite was the case for the 2016 event^56^, likely exacerbating heat stress. Both the 2010/11 and the 2016 event were prolonged marine heatwaves that lasted for several months, but heat stress peaked earlier (between January and early March^30^) in 2010/11 compared to 2016 (April-May). Bleaching patterns across these two events suggest that northern WA is particularly at risk of bleaching during strong El Niño years, whereas central-to-southern WA is vulnerable during strong La Niña years^32^. Rottnest Island could be particularly susceptible to frequent bleaching since it is the only site that bleached during both events, which threatens the potential of this high-latitude site to serve as climate change refuge^42^. Moreover, our findings highlight that regional-scale mass bleaching may now occur throughout WA during both El Niño and La Niña events^32^. As El Niño Southern Oscillation events will likely become more frequent and intense with continued climate change^36^, these findings have significant implications for the future resilience of coral reefs in WA.

## Electronic supplementary material

Supplementary Information
