# Supplementary material for: Marine heatwave causes unprecedented regional mass bleaching of thermally resistant corals in northwestern Australia
Source: Sci Rep. 2017 Nov 3;7:14999. doi: 10.1038/s41598-017-14794-y (PMC5670227; doi:10.1038/s41598-017-14794-y)
Supplement: Supplementary file 1 — Supplementary Information [file 41598_2017_14794_MOESM1_ESM.pdf]

Marine heatwave causes unprecedented regional mass bleaching of thermally resistant corals in northwestern Australia

Morane Le Nohaïc[1], Claire L. Ross[1], Christopher E. Cornwall[1], Steeve Comeau[1], Ryan Lowe[1,2], Malcolm T. McCulloch[1,2] and Verena Schoepf[1,2,*]

1. ARC Centre of Excellence for Coral Reef Studies, UWA Oceans Institute and School of Earth Sciences, The University of Western Australia, Perth, WA, Australia
2. The Western Australian Marine Science Institution, Perth, WA, Australia

*Corresponding author. Email: verena.schoepf@uwa.edu.au

Supplementary Information

Supplementary Tables

Supplementary Table S1. Abundance of health categories for all hard coral genera at all sites (except Bremer Bay) at the first and second survey time point. Data represent the mean percentage (± SE) of total live coral cover. Sample size is 4-6 transects. UB = unbleached, M = moderately bleached, S = severely bleached, D = dead (see Methods for details).

**Site**	**Montgomery Reef**	**Cygnet Bay**				**Ningaloo Reef**		**Rottnest Island**	
		**Subtidal**		**Intertidal**					
**Survey time point**	**2**	**1**	**2**	**1**	**2**	**1**	**2**	**1**	**2**
***Acropora* UB**	5.9 ± 1.7	87.2 ± 5.2	2.8 ± 1.8	65.6 ± 12.1	9.8 ± 4.7	18.4 ± 7.6	29.5 ± 5.1	40.9 ± 17.6	31.8 ± 20.1
***Acropora* M**	0.8 ± 0.3	1.7 ± 1.5	2.1 ± 1.0	0.2 ± 0.2	16.6 ± 3.4	14.7 ± 7.9			
***Acropora* S**	1.4 ± 0.6		79.6 ± 4.8	0.8 ± 0.6	41.3 ± 9.3	4.1 ± 4.1			
***Acropora* D**	5.5 ± 1.3		14.9 ± 3.6	4.1 ± 4.1	11.0 ± 3.7	23.7 ± 10.9	66.6 ± 4.9	0.04 ± 0.04	
***Caulastrea* S**									0.03 ± 0.03
***Caulastrea* D**	0.1 ± 0.1								
***Dipsastraea* UB**	0.4 ± 0.1	0.1 ± 0.05	0.1 ± 0.04	0.2 ± 0.1	0.1 ± 0.1	1.4 ± 1.4			0.03 ± 0.03
***Dipsastraea* M**	1.2 ± 0.2	0.03 ± 0.03	0.04 ± 0.03			0.6 ± 0.5			0.03 ± 0.3
***Dipsastraea* S**	1.4 ± 0.2	0.05 ± 0.05	0.5 ± 0.3		0.1 ± 0.1				

***Dipsastraea* D**	0.3 ± 0.1		0.1 ± 0.1		0.3 ± 0.2		0.2 ± 0.1		
***Favites* UB**	0.6 ± 0.4	0.1 ± 0.1	0.02 ± 0.02	0.4 ± 0.2		18.9 ± 11.5		0.2 ± 0.1	0.7 ± 0.4
***Favites* M**	2.5 ± 1.0					0.7 ± 0.7	0.01 ± 0.01		0.04 ± 0.03
***Favites* S**	3.5 ± 1.9	0.1 ± 0.1	1.0 ± 0.7	0.1 ± 0.05	0.1 ± 0.1				
***Favites* D**			0.5 ± 0.4						0.2 ± 0.2
***Fungia* UB**		0.1 ± 0.1	0.2 ± 0.2						
***Fungia* S**	0.03 ± 0.03								
***Galaxea* UB**	1.4 ± 0.5								
***Galaxea* S**	7.2 ± 0.7				1.2 ± 1.2				
***Galaxea* D**	2.1 ± 1.1				0.9 ± 0.9				
***Goniastrea* S**	0.1 ± 0.1								
***Goniopora* UB**	0.4 ± 0.4			0.6 ± 0.6					
***Goniopora* M**	0.2 ± 0.2								
***Goniopora* S**	0.3 ± 0.2		0.1 ± 0.1	4.9 ± 4.9	1.3 ± 1.2				
***Goniopora* D**	0.4 ± 0.4		0.05 ± 0.05		1.7 ± 1.7				
***Herpolitha* M**	0.2								

	± 0.2					
***Herpolitha* S**	0.03 ± 0.03					
***Lobophyllia* UB**		0.05 ± 0.05		4.1 ± 3.8	1.1 ± 1.1	0.4 ± 0.4
***Lobophyllia* M**	0.3 ± 0.3					
***Lobophyllia* S**	1.3 ± 0.7		0.3 ± 0.1		0.5 ± 0.5	
***Lobophyllia* D**			0.02 ± 0.02		0.2 ± 0.2	
***Millepora* UB**				1.1 ± 1.1		
***Montastrea* UB**				0.4 ± 0.3		
***Montastrea* M**	0.09 ± 0.06					
***Montastrea* S**	0.3 ± 0.03					
***Montipora* UB**	9.4 ± 0.5		0.4 ± 0.3	2.1 ± 1.1		0.1 ± 0.1
***Montipora* M**	8.2 ± 2.5		0.1 ± 0.1			
***Montipora* S**	10.8 ± 2.5		0.5 ± 0.5			
***Montipora* D**	0.7 ± 0.7		0.3 ± 0.3			
***Pavona* UB**			0.04 ± 0.04	1.1 ± 0.7		

***Pavona* M**					0.1 ± 0.1				
***Pavona* S**			0.1 ± 0.04	0.3 ± 0.8	0.7 ± 0.6				
***Platygyra* UB**		0.04 ± 0.03	0.05 ± 0.05	0.3 ± 0.2		3.6 ± 2.8	0.8 ± 0.8	7.7 ± 7.4	
***Platygyra* M**	3.7 ± 2.2	0.05 ± 0.05	0.1 ± 0.1						0.1 ± 0.1
***Platygyra* S**	3.7 ± 1.9	0.03 ± 0.03	0.1 ± 0.1		0.5 ± 0.5				
***Platygyra* D**	0.3 ± 0.2				0.1 ± 0.1				
***Pocillopora* UB**		0.9 ± 0.7	0.03 ± 0.03					46.7 ± 15.8	37.4 ± 15.6
***Pocillopora* M**			0.1 ± 0.1		0.04 ± 0.04			3.7 ± 1.3	28.0 ± 14.3
***Pocillopora* S**			0.7 ± 0.4						0.5 ± 0.4
***Pocillopora* D**			0.2 ± 0.1					0.4 ± 0.2	0.7 ± 0.4
***Porites* UB**		0.3 ± 0.3	0.8 ± 0.6	8.9 ± 3.6	2.6 ± 2.2	1.8 ± 0.8	0.1 ± 0.1		
***Porites* M**	0.3 ± 0.3		1.3 ± 1.2		2.0 ± 1.2	0.5 ± 0.5			
***Porites* S**	1.0 ± 0.8		0.7 ± 0.2	0.3 ± 0.3	1.5 ± 0.6				
***Porites* D**			0.2 ± 0.2		0.1 ± 0.1				
***Seriatopora* UB**	0.6 ± 0.6	0.7 ± 0.4							

***Seriatopora* M**		2.7 ± 2.4							
***Seriatopora* S**	1.3 ± 0.6	0.02 ± 0.02	0.4 ± 0.2	0.3 ± 0.3					
***Seriatopora* D**	1.8 ± 1.3		0.1 ± 0.1						
***Stylophora* UB**		0.05 ± 0.05		0.8 ± 0.8			0.1 ± 0.1		
***Stylophora* S**		0.5 ± 0.4	1.0 ± 0.8	0.2 ± 0.2					
***Trachyphyllia* M**				0.3 ± 0.3					
***Trachyphyllia* S**					0.1 ± 0.1				
***Tubipora* UB**	0.6 ± 0.05								
***Turbinaria* UB**	0.05 ± 0.5	1.1 ± 0.6	0.3 ± 0.3	0.9 ± 0.5	0.2 ± 0.2	0.7 ± 0.7			
***Turbinaria* M**	0.1 ± 0.1	0.1 ± 0.1	1.5 ± 1.5						
***Turbinaria* S**	0.2 ± 0.2		0.6 ± 0.6						
***Turbinaria* D**			0.3 ± 0.3						
**Unkown UB**	4.9 ± 2.0	3.6 ± 2.8		1.9 ± 0.9		5.3 ± 3.3	1.1 ± 0.7	0.3 ± 0.3	
**Unknown M**	0.8 ± 0.2	0.2 ± 0.2			0.6 ± 0.5	5.0 ± 4.6	0.2 ± 0.1		
**Unknown S**	5.8 ± 2.0			0.05 ± 0.05	5.1 ± 3.8				0.1 ± 0.1

**Unknown D**	8.4 ± 1.6	1.6 ± 1.1

Supplementary Figures

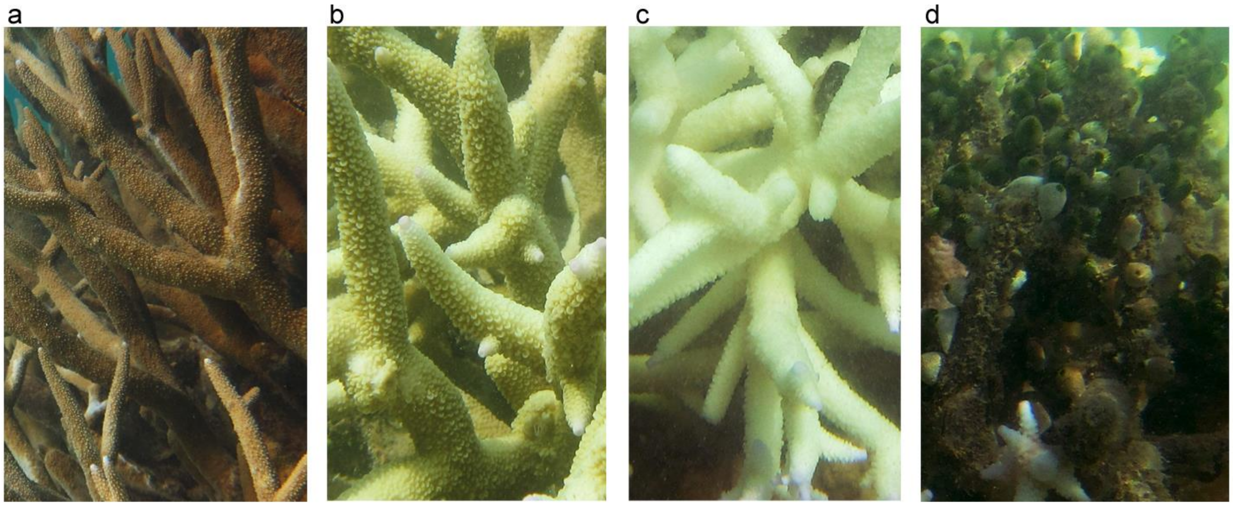

Supplementary Figure S1. Photos of branching *Acropora* corals representing the four health categories: (a) UB = Unbleached, (b) M = Moderately bleached, (c) S = Severely bleached, and (d) D = Dead. Photos from M. Le Nohaïc and C. E.Cornwall.
